# Erupted frothy xenoliths may explain lack of country-rock fragments in plutons

**DOI:** 10.1038/srep34566

**Published:** 2016-11-02

**Authors:** Steffi Burchardt, Valentin R. Troll, Harro Schmeling, Hemin Koyi, Lara Blythe

**Affiliations:** 1 Department of Earth Sciences, Uppsala Universitet, Villavägen 16, 75236 Uppsala, Sweden; 2 Faculty of Earth Sciences, J. W. Goethe Universität, Altenhöferallee 1, 60438 Frankfurt am Main, Germany

## Abstract

Magmatic stoping is discussed to be a main mechanism of magma emplacement. As a consequence of stoping, abundant country-rock fragments should occur within, and at the bottom of, magma reservoirs as “xenolith graveyards”, or become assimilated. However, the common absence of sufficient amounts of both xenoliths and crustal contamination have led to intense controversy about the efficiency of stoping. Here, we present new evidence that may explain the absence of abundant country-rock fragments in plutons. We report on vesiculated crustal xenoliths in volcanic rocks that experienced devolatilisation during heating and partial melting when entrained in magma. We hypothesise that the consequential inflation and density decrease of the xenoliths allowed them to rise and become erupted instead of being preserved in the plutonic record. Our thermomechanical simulations of this process demonstrate that early-stage xenolith sinking can be followed by the rise of a heated, partially-molten xenolith towards the top of the reservoir. There, remnants may disintegrate and mix with resident magma or erupt. Shallow-crustal plutons emplaced into hydrous country rocks may therefore not necessarily contain evidence of the true amount of magmatic stoping during their emplacement. Further studies are needed to quantify the importance of frothy xenolith in removing stoped material.

The emplacement mechanism of large volumes of magma into the Earth’s crust to form magma reservoirs (and plutons) has been puzzling petrologists and structural geologists since the infancy of modern geology (cf. the “space problem”^1^,^2^,^3^,^4^). For the upper crust, three main emplacement mechanisms are generally discussed^5^: (1) roof uplift^6^, (2) floor subsidence^7^,^8^, and (3) magmatic stoping^1^. Stoping is originally defined as the continued fracturing of the roof and walls of a magma body causing the detached country-rock fragments to move in their host magma^1^. The main process driving the detachment of wall rocks is considered magma-driven fracturing caused by e.g. thermal stresses (e.g. refs 1, 9 and 10). Hence, magmatic stoping should occur preferentially in the upper crust where temperature differences between the country rock and magma are largest^5^. Outcrop features that frequently occur in exposed plutons worldwide, and that are commonly cited as evidence for stoping, include (a) country-rock xenoliths and partly-detached roof pendants, (b) mixed xenolith populations, (c) local absence of contact-aureole rocks along pluton walls, (d) stepped intrusive contacts, and (e) host-rock structures that are discordant to intrusive contacts (e.g. refs 5 and 11, 12, 13, 14). However, the widespread observation of the above features is in contrast to the general lack of significant volumes of xenoliths in many plutons, which rarely exceeds 1% of outcropping pluton volumes (e.g. ref. 15). Another argument that is used against the efficiency of magmatic stoping is that the amount of crustal contamination recorded in plutons frequently falls short of that expected if bulk assimilation of the stoped material is assumed (ref. 13 and references therein). This is further complicated by the limited ability of magma to melt of country rock, which usually does not exceed 30–35 vol.% due to a magma’s energy budget^16^, even though selective assimilation of xenolithic material is a far more likely to occur^17^,^18^. Therefore, the efficiency of magmatic stoping to create space for magma in the crust and contribute to upward magma emplacement is the main focus of discord between those who fundamentally question the principle of stoping *per se*^13^,^19^ and those who advocate stoping as a significant mechanism of magma emplacement in the brittle crust^10^,^18^,^20^. The outcome of this discussion will affect our thinking on a wide range of fundamental problems in Earth sciences, including magmatic recycling of crustal material to “distill” continental crust (e.g. ref. 21), crustal thickening through magmatic underplating (e.g. ref. 22), increased volcanic explosivity through crustal volatiles (cf. refs 23 and 24), and the formation of contact-metamorphic mineral deposits (e.g. ref. 25).

The lack of abundant country-rock xenoliths in plutons is a key point in the ongoing controversy on magmatic stoping. Traditionally, stoped material has been thought to often sink to the bottom of the host magma reservoir where it should accumulate in xenolith graveyards or become gradually assimilated (e.g. refs 17 and 26). However, such graveyards that would manifest as magmatic breccia horizons are extremely scarce, both at the bottom of plutons and within them^13^. Additionally, the efficiency of sinking of xenoliths to the bottom of a magma reservoir is limited by the amount of liquid magma present at any given time, which may be small considering incremental magma injection and the presence of crystal mushes and partly solidified mush domains^27^,^28^,^29^. While the aspects of upward emplacement of magma and space generation associated with magmatic stoping are beyond the scope of this study, we present petrographic and numerical modelling evidence regarding the potential whereabouts of the missing xenoliths. We propose that at least a portion of the stoped material might be found in volcanic products instead. There “frothy” xenoliths (“xeno-pumice”^30^) may represent the remnants of stoped material that experienced volatile exsolution during partial melting while entrained in magma. Volatile exsolution and associated density decrease of xeno-pumice may allow to efficiently remove xenolithic material from the plutonic record. Below, we will describe a selection of such frothy xenolith samples and analyse the effect of partial melting and density decrease on xenolith buoyancy while emersed in magma though thermomechanical numerical models.

## Results

### The missing link?- xenoliths in volcanic rocks

**Figure 1:**
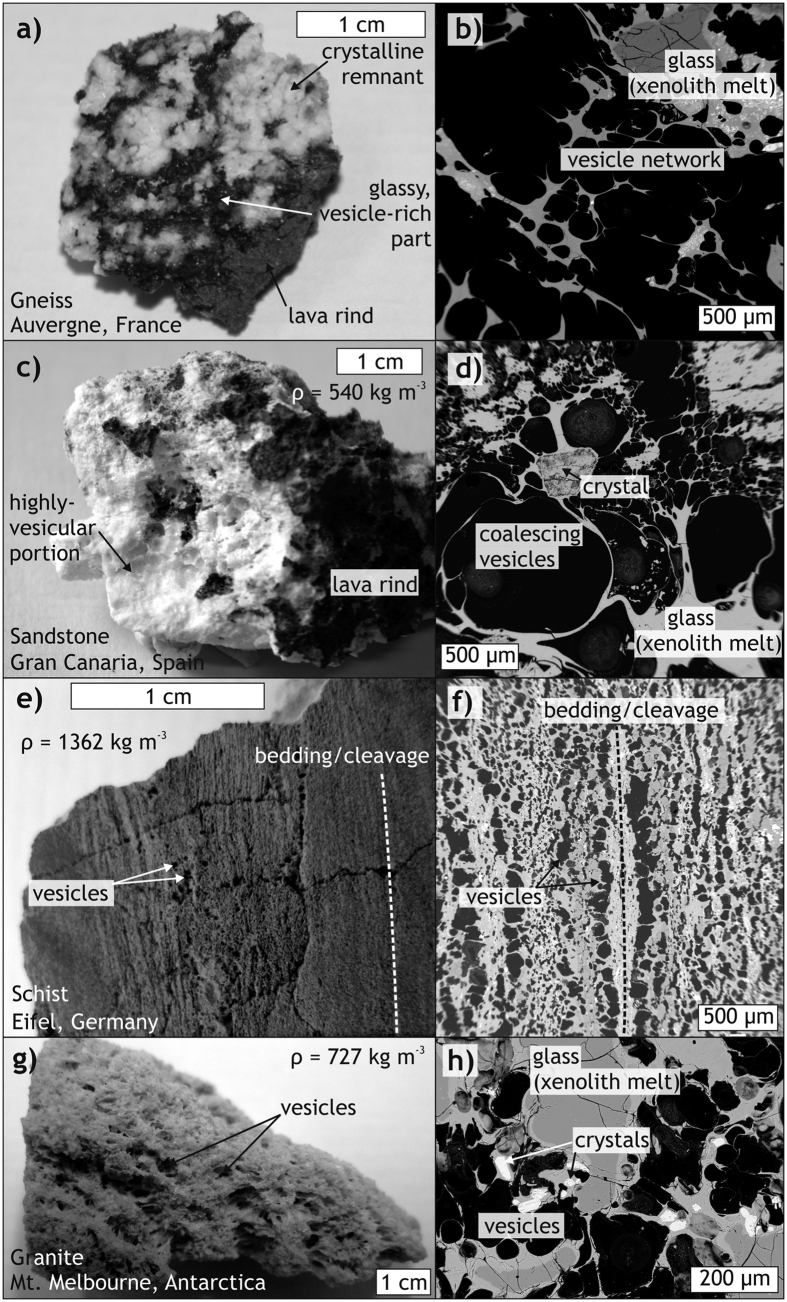
Appearance and structure of examples of frothy xenolith fragments. Densities have been determined using the Archimedes method after Kueppers *et al*.^57^. Errors are estimated to be approximately 5%. Left column: sample photographs. Right column: Scanning Electron Microscope (SEM) images. (**a**,**b)** Partially melted and vesiculated gneiss fragment enclosed in phonolitic lava from the Auvergne, France. (**c,d**) Vesicular marine arkose enclosed in basaltic scoria from Gran Canaria, Canary Islands, Spain^34^. (**e,f)** Vesiculated schist from the Eifel, Germany. Vesicles form preferentially along the bedding/cleavage planes. (**g,h)** Frothy former granite erupted from Mt. Melbourne, Antarctica.

**Figure 2:**
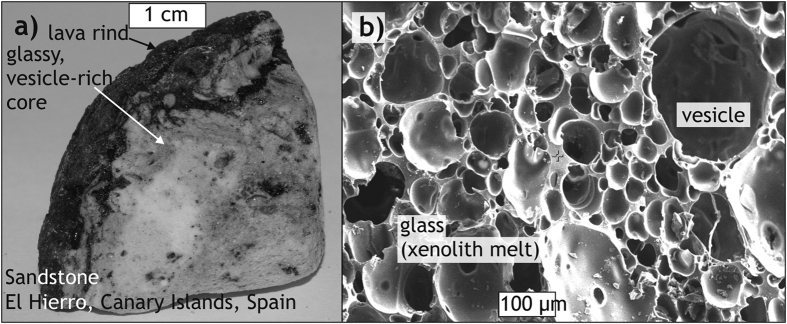
Appearance and structure of a frothy sandstone xenolith sample from the 2011 offshore eruption at El Hierro, Canary Islands, Spain (cf. ref. 30). (**a)** Sample photograph. (**b)** SEM image of the sample’s pervasive vesiculation.

Frothy xenoliths or “xeno-pumice” (Fig. 1) of variable depth assemblages and originating from virtually all crustal rock types occur in many extrusive rocks from subduction-zone, ocean-island, and continental intra-plate settings (e.g. refs 23, 24 and 31, 32, 33, 34, 35, 36). Most recently, during the early stages of the 2011 submarine eruption south of El Hierro, Canary Islands, Spain, such frothy xenoliths were found floating on the ocean surface (i.e. their density was ≤1; Fig. 2 30). Their mineral assemblage, sedimentary relicts, and the occurrence of nannofossils showed that these pumice-like rocks were of sedimentary origin and entrained by the ascending magma^30^,^37^. Intense seismicity at the level of the sub-island sedimentary strata prior to the eruption coupled with surface deformation patterns that indicate inflation at this depth level^38^,^39^,^40^ may be interpreted as magma-driven fracturing, i.e. magmatic stoping.

In addition to the floating xeno-pumice from El Hierro, similar frothy xenoliths in eruptive products have been reported from several continents (including Antarctica) and cover sandstone, granite, schist, and gneiss lithologies that originated from various depths beneath these volcanoes (Figs 1 and 2). The frothy texture of these xenoliths results from partial melting and associated volatile exsolution and inflation while subjected to magmatic temperatures^17^,^30^,^34^,^35^,^36^,^37^. Vesicles within these samples record bubble nucleation either distributed throughout the rock (e.g. Figs 1a,d and 2), or preferentially along anisotropies, such as foliation planes (Fig. 1f). Vesicle nucleation, growth, and coalescence produced vesicle networks at various stages of connectivity (e.g. Fig. 1b,d,f,h) that record volume increase of the xenoliths and volatile transport into the surrounding host magma, and thus caused a decrease in xenolith density (Fig. 1). These processes can be reproduced under laboratory conditions, using heating and decompression, to produce structures that strikingly resemble those of the natural xenolith samples^35^,^41^. Frothy xenoliths may thus represent some of the missing stoped material that was expelled from the host magma reservoir and conduits as a result of heating, devolatilisation, inflation, and associated density decrease during magma storage within the crust.

### Sink or swim?- Modelling the effect of xenolith melting and density decrease

In order to analyse xenolith behaviour as a consequence of the physical changes caused by partial melting and devolatilisation while entrained in magma, we ran a series of finite differences models. The models aim to explore whether the process of frothing (vesiculation) allows xenoliths to escape sinking once they have dropped into a magma reservoir. Our models resemble the setup of analogue experiments performed by McLeod and Sparks (1998)^42^ who simulated the sinking and melting of spherical xenoliths in a hotter magma. Specifically, we consider a hydrothermally altered, older granite xenolith from the aureole entrained into dry granitic magma (see Methods section) to simulate a scenario that may occur during pluton emplacement in the continental crust (cf. ref. 9). Our models simulate the effect of a mild density decrease on the dynamics of xenolith sinking during melting. Because our approach cannot produce actual vesiculation, we account for this process by considering a temperature-triggered phase transition from solid xenolith to a melt-vesicle suspension with a density lower than that of dry xenolith melt (ca. 91%; see Table 1). In successive models, we then varied the initial xenolith temperature, and the viscosity of the xenolith melt-vesicle suspension.

**Figure 3:**
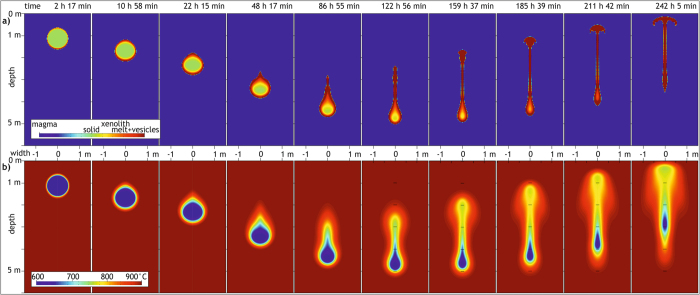
Model results of thermomechanical processes associated with a xenolith sinking in a granitic magma chamber. Physical parameters are listed in Table 1. The xenolith is assumed to be a hydrothermally altered granite already heated by the intruding magma. Relevant parts of the 5 m wide and 10 m deep model are displayed for selected time steps. Beyond these selected parts, no changes in the compositional and temperature fields occur. (**a)** Compositional field (resolution of Finite Differences grid 2.5 × 2.5 cm^2^), illustrating the different phases and the phase transition from solid to molten xenolith. (**b)** Temperature distribution (resolution 1.25 × 1.25 cm^2^).

In a first model, the xenolith starts to sink slowly, whilst progressively heating up and melting on all exterior sides at the same rate, producing a shell of melt-vesicle suspension that surrounds a still solid core (Fig. 3). The host magma, in turn, is cooled preferentially above the xenolith, but not to a degree that it would solidify (<800 °C) or keep the xenolith from moving. The xenolith melt-vesicle suspension is sheared off the sinking xenolith core and accumulates along its top (cf. ref. 42). This way, the xenolith core is progressively exposed to new, high-temperature host magma, causing a gradual acceleration of melting rates at this stage. Eventually, the xenolith melt-vesicle suspension detaches from the xenolith core as a small diapir and, rises towards the top of the magma reservoir. After ca. 5.5 days, the remaining xenolith core ceases to sink also, and a remnant of the still solid core is present, but is now so small in size that it is dragged upwards by the surrounding xenolith melt-vesicle suspension. After ca. 10 days, virtually all core material is partially molten, and vesiculation is pervasive. Hence, downward movement of the xenolith is only an early stage phenomenon in the model.

**Figure 4:**
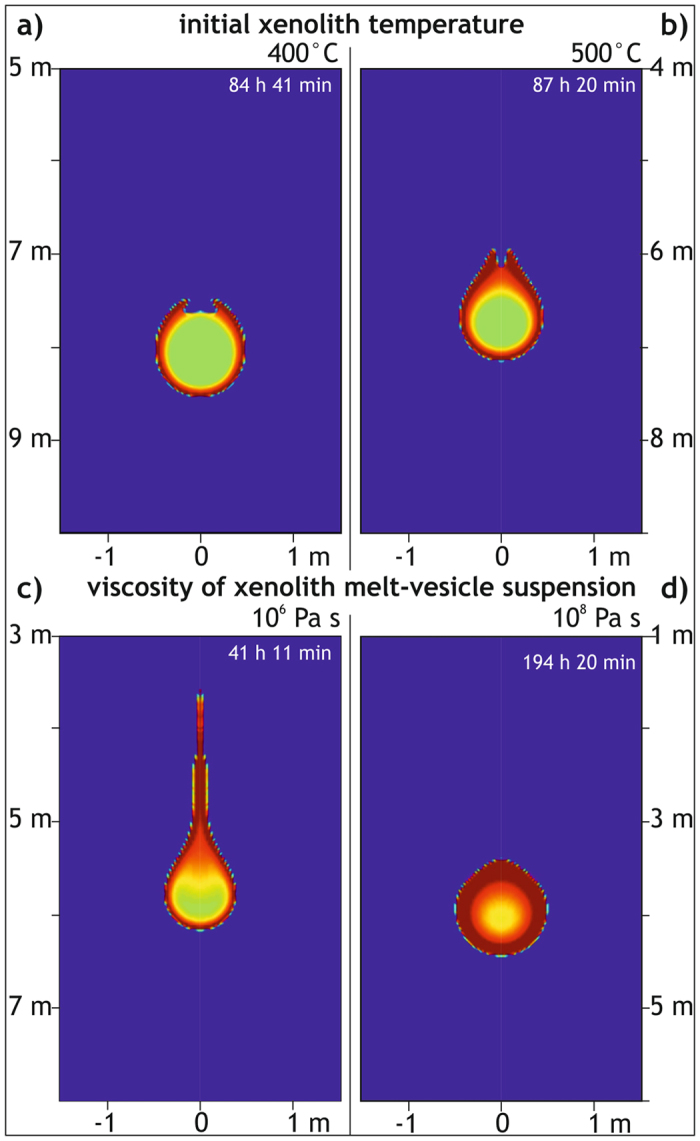
Influence of basic parameters on model results of thermomechanical processes associated with a xenolith sinking in a granitic magma chamber. Relevant parts of the 5 m wide and 10 m deep model are displayed for selected time steps. Beyond these selected parts, no changes in the compositional and temperature fields occur. Physical parameters that were changed in comparison to the model illustrated in Fig. 2. include the initial xenolith temperature: (**a)** T = 500 °C, (**b)** T = 400 °C and the viscosity of the xenolith melt: (**c)** 10^8 ^Pa s, (**d)** 10^6 ^Pa s.

Using a lower starting temperature of the xenolith, results in a predictably longer xenolith melting time. In this case, melting along the xenolith margin starts as a thinner layer, and detaches as two thin lobes from the xenolith that later unite into a rising melt diapir (Fig. 4a,b). Consequently and somewhat counter-intuitively, the colder and denser xenolith interior initially sinks faster and is therefore continuously exposed to new high-temperature host magma due to melt detachment, resulting in more efficient melting and vesiculation of the xenolith per unit time and thus causes ultimate floating after a shorter time.

In turn, in a model where the viscosity of the xenolith melt-vesicle suspension is of the same viscosity as the surrounding granitic magma, the xenolith melt does not detach from the solid xenolith core but envelopes it. Strikingly, this causes (1) slower sinking of the xenolith, (2) slower melting and vesiculation of the xenolith interior, (3) rising of the remaining xenolith core at a lower melt fraction, and (4) a higher ascent velocity of the xenolith once it starts to rise (Fig. 4c). In comparison, a decrease in the viscosity of the xenolith melt-vesicle suspension speeds up detachment of the new melt from the solid xenolith core (Fig. 4d).

Whether a xenolith will become buoyant enough to float after initial sinking, is therefore determined by the physical properties of the xenolith, as well as its size relative to the vertical dimension of the liquid part of the magma reservoir. Xenoliths that are not sufficiently molten and inflated before reaching the reservoir floor may get trapped there by cumulates and may eventually become restite material. It is, however, conceivable that such xenoliths at the reservoir bottom vesiculate with time and subsequently liberate themselves from e.g. unconsolidated mush like an Alka Seltzer tablet rising from the bottom of a glass of water.

## Discussion

**Figure 5:**
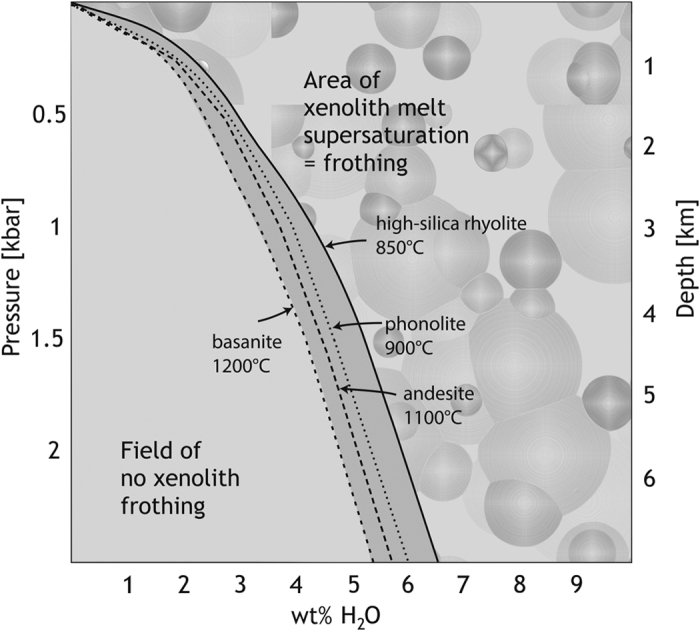
Pressure conditions for frothing of crustal xenoliths entrained in different types of magma as a function of xenolith water content. The curves display H_2_O solubility for the stated melt composition as a function of pressure and wt% H_2_O. Modified from Holloway and Blank^58^.

Although it is as yet not possible to give a quantitative estimate of the percentage of frothed xenoliths among erupted xenolithic material, the occurrence of frothed crustal xenoliths in volcanic rocks in a wide variety of geodynamic settings (Figs 1 and 2) demonstrates that some of the xenoliths have been erupted instead of preserved in the intrusive record. Using a set of thermomechanical models, we explored whether the process of frothing would allow xenoliths to escape sinking once they have dropped into a magma reservoir. Our modelling results demonstrate that a mild density decrease of xenoliths as a result of devolatilisation during partial melting is probably sufficient in many magmatic systems to stop xenolith sinking and make them float instead. Although the parameter space we consider in our models is limited and targeted to a specific scenario relevant for the emplacement of some granitic plutons into continental crust (cf. Table 1), our models in fact simulate rather unfavourable conditions, such as a high-viscosity host magma, a xenolith with relatively high melting temperature and a mild density decrease only. Future modelling will have to systematically simulate the effect of a wider range of magma and xenolith properties relevant for different settings. However, our models demonstrate that xenoliths may be able to rise, and potentially escape, due to devolatilisation. In nature, more forceful reactions compared to our models, e.g. faster melting, stronger volatile exsolution, and/or faster ascent may be expected for magmas with higher temperatures or lower viscosities, for xenolith lithologies with lower melting temperatures and/or for a larger density contrast between magma and vesiculated xenoliths. Moreover, in nature, chemical reactions between the xenolith and the host magma, as well as differential melting of different mineral phases in the xenolith, likely modify the details of the melting process, but cannot be accounted for in our models. The described process of frothing can generally be expected to be most pronounced at low pressures and when the xenolith is volatile-rich (Fig. 5). These conditions are often met in upper–crustal magma reservoirs and conduits where different types of magma intrude into sedimentary, or into altered igneous or metamorphic rocks containing up to several wt.% of volatiles (cf. Fig. 5; e.g. ref. 43). Once a volatile-bearing crustal rock gets entrained into a magma, the xenolith magma will be oversaturated in volatiles and vesicles will form^44^. In addition, processes such as xenolith fragmentation and expansion due to thermal stresses and volatile dissolution (cf. refs 9, 17, 35 and 41) would aid and accelerate xenolith melting. Although our models do not consider these processes and only take a mild decrease in density into account, large effects for the xenolith can be anticipated, i.e. the xenoliths may start to float after an initial phase of sinking.

While our study does not resolve how magmatic stoping may contribute to generating space during magma emplacement, our results provide a relevant new argument on the apparent lack of xenolithic material in plutons. We show that xenoliths do not necessarily have to accumulate somewhere within, or sink to the bottom of, a magma reservoir and build a xenolith graveyard. Plutons might be, by the very nature of the system, the wrong place to look for a large amount of xenoliths. Instead, many xenoliths may rise within their host magma, as demonstrated by our models. Xenolith remnants, melt, and the released xenoliths may then concentrate in the cupola of a magma reservoir (e.g. ref. 45). Although our models cannot simulate mixing of xenolith melt and host magma, it is conceivable that the volatile exsolution from xenoliths adds to the volatile budget of the magma^23^,^24^,^39^ and may therefore trigger volcanic eruptions that remove xenoliths from the plutonic record (Fig. 1). Evidence for this hypothesis is provided by the occurrence of highly heterogeneous partial xenolith melts (e.g. refs 46 and 47) and the frequently higher degrees of crustal contamination of volcanic rocks compared to their plutonic equivalents (e.g. ref. 48). Frothy xenoliths in extrusive rocks worldwide (e.g. refs 23, 30, 31, 32, 34 and 49) may hence be regarded as “snapshots” of this process. Indeed, highly-contaminated, low-density magmas that are usually the first to erupt as part of larger, chemically zoned eruptions (cf. refs 31, 46, 47, 48 and 50) may in part reflect such assimilated xenoliths in the cupola region of a larger magma reservoir.

In addition, vesiculation during xenolith ascent will accelerate disintegration of xenoliths, so not all xenolithic material needs to be melted or ejected to “disappear” (cf. ref. 51). Approximately 30–35 vol.% of country rock can be effectively melted by a magma^16^, but if solid material breaks down and is dispersed in the host magma (cf. ref. 52), assimilated volumes may in fact be much higher. This is evident from the common occurrence of xenocrysts in lavas and pyroclastic deposits (e.g. refs 53 and 54). Under normal circumstances, careful sample preparation would avoid mineral clots, larger-than-usual crystals, or textural anisotropies (restites), hence, probably masking the actual amount of assimilation of crustal material in many sample analyses.

To synthesise, the combined observations on frothy xenolith samples and our numerical model results imply that the plutonic record may simply not contain the complete amount of xenolith evidence. A portion of xenoliths may in fact have risen towards the magma-reservoir roof from where they were eventually erupted, either as frothy xenoliths or in form of assimilated solids and liquids in lava and pyroclastic deposits.

## Methods

We used a two-dimensional Finite Differences code (FDCON^55^,^56^) to model the thermomechanical aspects of this system. We modelled a two-phase compositional system including a temperature-triggered phase transition from solid xenolith material to partially molten, low-density, vesiculated xenolith (vesicle-melt suspension), including the latent heat of fusion. Here, a volatile-rich, e.g. hydrothermally altered, ‘older’ xenolith (e.g. wet granite) is enclosed in a ‘younger’ granitic magma. A stream function formulation is used to solve the equations of conservation of mass and momentum. The equation of conservation of composition of the granitic magma and the xenolith is solved applying the “marker-in-cell method”^56^.

Physical properties of the materials used are taken from McLeod and Sparks^42^ (Table 1) and adjusted to account for the effect of volatiles in the hydrothermally altered granitic xenolith. Even though a true vesiculation of the xenolith (producing melt + vesicles), including volumetric expansion, cannot be reproduced, our model accounts for the effect of vesiculation of the xenolith by assuming a density decrease during partial melting producing a vesicle-melt suspension with a density below that of dry granite melt. Since materials in FDCON are defined as incompressible, the xenolith-melt density is limited to 2000 kg m^−3^ (i.e. 91% of ρ_melt(granite)_), a rather conservative value compared to the dramatic density decrease measured in our natural xenoliths (Fig. 1).

The model simulates a xenolith (circular, diameter 1 m, T_initial_ = 600 °C) which is placed with its centre at a depth of 1 m into a section of a granitic magma reservoir (rectangular, 5 m wide, 10 m deep; T = 900 °C). The model boundaries are isothermal, enable free slip of material, and represent mirror planes to minimise boundary effects and processing time. The geometrical scaling of the models takes into account the size of natural xenoliths in magma reservoirs that range from cm to tens of metres. However, as stoping requires a certain magma volume, the size of the reservoir and the resulting xenoliths are defined through the surface area of the active interface between magma reservoir and xenolith.

## Additional Information

**How to cite this article**: Burchardt, S. *et al*. Erupted frothy xenoliths may explain lack of country-rock fragments in plutons. *Sci. Rep.* **6**, 34566; doi: 10.1038/srep34566 (2016).

## Figures and Tables

**Table 1:** Physical properties of modelled materials.

Granitic magma chamber	
ρ_m_ density	2200 kg m^−3^
μ_m_ viscosity	10^7^ Pa s
T initial temperature	900 °C
T_m_ melting temperature (solidus)	800 °C
Granite xenolith	
ρ_s_ density(solid)	2600 kg m^−3^
ρ_m_ density(melt)	2000 kg m^−3^
μ_s_ viscosity(solid)	10^11^ Pa s
μ_m_ viscosity(melt)	10^7^ Pa s
T initial temperature	600 °C
T_m_ melting temperature	700 °C
All materials	
κ thermal diffusivity	8 10^−8^ m^2^ s^−1^
C heat capacity	1340 J kg^−1^ °C^−1^
L melting enthalpy	2.93 10^5^ J kg^−1^

Parameters are derived from McLeod and Sparks^42^.

Density of the molten xenolith takes into account the effect of devolatilisation.
